# The Midwives' Registration Bill

**Published:** 1898-04-09

**Authors:** 


### THE MIDWIVES' REGISTRATION BILL.

So far as we are concerned as a profession, the proposed Bill bristles with points of interest. But so far as it touches the interests of the public, the first and chief point is, Will it do what its promoters profess to be the very object of its introduction? Will it put a stop to the practice of midwifery by ignorant and unregistered women? As the Bill stands at present, there can be no doubt that it will do nothing of the sort. There is not a word in it to prevent them doing as they like so long as they do not assume certain titles.

Dr. Cullingworth says, with great force, that as things stand at present, midwives "may be grossly incompetent, they may spread puerperal fever broadcast, they may be drunken, they may take upon themselves to give medicines, perform operations, and undertake duties which can only be safely undertaken by a fully and properly qualified doctor." The obvious moral would seem to be that something should be done to check all these practices. We look, however, in vain for anything in the Bill by which these evils are to be interfered with; and, when we ask why, we are told that Parliament would never consent to any restraint upon practice, and that any attempt to introduce such restraint would wreck the Bill. We are not quite so sure of this. Give Parliament a good case, and Parliament will go a long way to reform an obvious abuse.

So far as the principle of interference with a trade is concerned, it is worth while to recall what happened in regard to the Steam Engines (Persons in Charge) Bill. This was not a case of restricting the assumption of a title, but requiring that persons in charge of engines and boilers should obtain a certificate of efficiency. It was a direct interference with the daily work of thousands of men; yet it passed the second reading.

A like Bill was again introduced last year, the second reading being moved on February 17th. This Bill proposed that it should not be lawful for any person to take charge of any boiler or engine to which the Bill applied unless he was qualified by a certificate of competence as required by the measure. This was a direct interference with practice. It would have taken the bread out of the mouths of those who could not obtain the certificate. Yet, notwithstanding that it was opposed, and that the Government declined to support it, the principle of the Bill was confirmed by 203 votes to 137.

With that before us, although, from other causes, the Steam Engines Bill did not pass, we hesitate to accept the statements made by the promoters of the Midwives Bill to the effect that the measure would inevitably be wrecked by the insertion of such a clause as should at least enable it to fulfil its purpose, namely, to prevent the practice of midwifery by ignorant and unsuitable persons. Parliament will certainly require some sort of assurance that the measure before it will remove the abuses against which it is professedly aimed. These assurances cannot be given as the Bill stands now.

